# Alkali–metal nickelates: catalytic cross-coupling, clusters and coordination complexes

**DOI:** 10.1039/d4cc03548h

**Published:** 2024-08-14

**Authors:** Andryj M. Borys, Eva Hevia

**Affiliations:** a Departement für Chemie, Biochemie und Pharmacie, Universität Bern 3012 Bern Switzerland andryj.borys-smith@unibe.ch eva.hevia@unibe.ch

## Abstract

Alkali–metal nickelates are a class of highly reactive heterobimetallic complexes derived from Ni(0)–olefins and polar organo-alkali–metal reagents. First reported over 50 years ago, it is only in recent years that these overlooked complexes have found formidable roles in sustainable catalysis and beyond. In this article, we will showcase the emerging catalytic applications of lithium nickelates and discuss the mechanisms by which these heterobimetallic complexes facilitate challenging cross-coupling reactions. We will also review the unique structure and bonding of alkali–metal nickelates, as interrogated by X-ray crystallography and complementary bonding analysis, and finally explore the diverse coordination and co-complexation chemistry of these heterobimetallic complexes.

## Abstract 2

Alkali–metal nickelates are a class of highly reactive heterobimetallic complexes derived from Ni(0)–olefins and polar organo-alkali–metal reagents.

## Graphical abstract

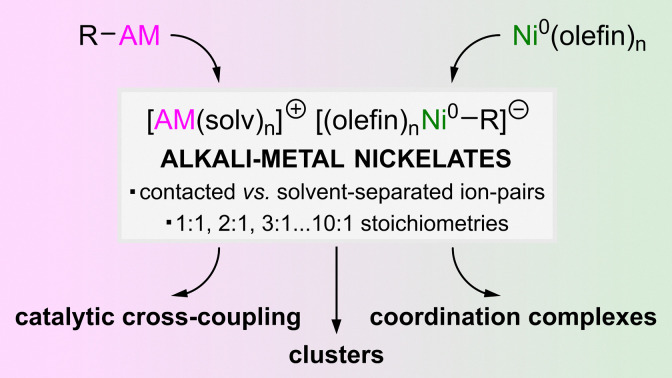

## 1. Introduction

**Scheme 1:**
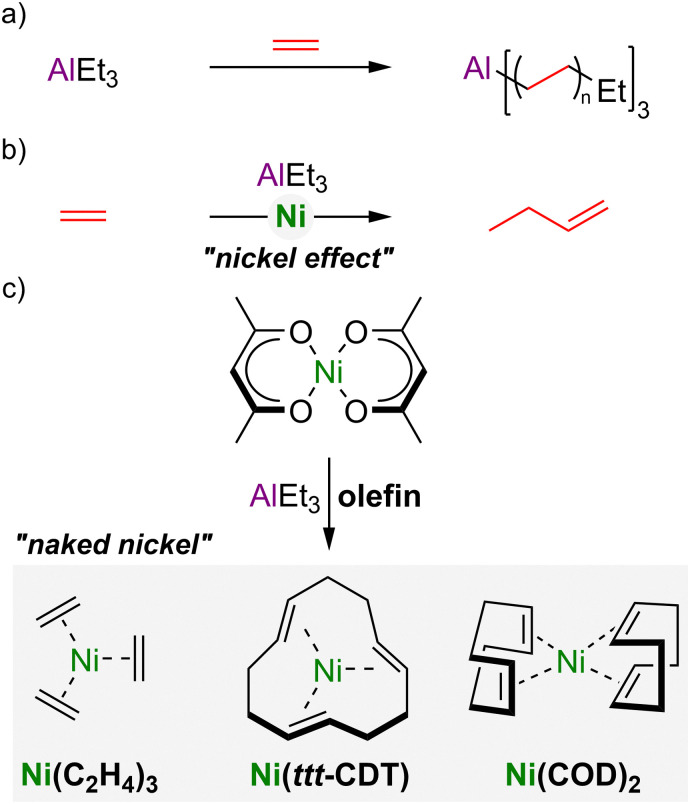
(a) Reaction of AlEt_3_ with ethylene to give long-chain trialkyl–aluminium compounds; (b) the “nickel effect” leading to ethylene dimerisation to give 1-butene. (c) Synthesis of Ni(0)–olefin complexes by treatment of Ni(acac)_2_ with AlEt_3_ in the presence of suitable olefins.

The synthesis of Ni(0)–olefin complexes by Wilke and co-workers represents a landmark in transition-metal and organometallic chemistry.^1–3^ The origin of this discovery is routed in the so-called “nickel effect”^4^ in the development of Zeigler catalysts, in which triethylaluminium reacts with ethylene at 100 °C under pressure to form long-chain trialkylaluminium compounds (Scheme 1(a)),^5^ which yields linear alkanes upon hydrolysis. Serendipitously, however, it was found that trace nickel salts present in the reaction autoclave led exclusively to 1-butene formation, the dimer of ethylene, formed through chain cleavage after each insertion step (Scheme 1(b)).^6^ Following systematic investigations, it was eventually realised that the treatment of Ni(acac)_2_ (where acac = acetylacetonate) with organoaluminium compounds in the presence of olefins enables the synthesis and isolation of “naked nickel” Ni(0)–olefin complexes (Scheme 1(c)), including Ni(C_2_H_4_)_3_, Ni(*ttt*-CDT) and Ni(COD)_2_ (where *ttt*-CDT = *trans*,*trans*,*trans*-1,5,9-cyclododecatriene and COD = 1,5-cyclooctadiene).^1–3^ To this day, Ni(COD)_2_ still represents the ubiquitous Ni(0) source, due to its widespread applications as a versatile precursor or (pre)catalyst, as well as its commercial availability or facile synthesis.^7,8^

In the years following the discovery of Ni(0)–olefin complexes, Wilke and co-workers at the Max-Planck-Institut für Kohlenforschung extensively investigated the interaction of these low-valent species towards a range of polar organometallics of the main-group, including organolithium and organoaluminium compounds.^9–13^ Here, the carbanion of the polar organometallic acts as strong σ-donating ligand and coordinates to the nickel centre, often with displacement of an olefin ligand, giving rise to heterobimetallic nickelate complexes. Numerous factors were found to impact the speciation of the resulting heterobimetallic nickelate, including the electronic properties of the carbanion, the reaction stoichiometry, identity of the secondary metal and its solvation. Whilst the broader synthetic and catalytic utility of heterobimetallic nickelates was unknown during these early studies, their extreme sensitivity and high reactivity was already well documented, as best illustrated by their ability to activate dinitrogen or cleave ethereal solvents.^14–16^ Despite this wealth of fundamental research, little of which has been published,^9^ there has been limited progress in the field since the late 1980s. Notably, no direct applications to catalysis were documented during this time and it is only in recent years that the resurgence of heterobimetallic nickelates arose.^17^

**Scheme 2:**
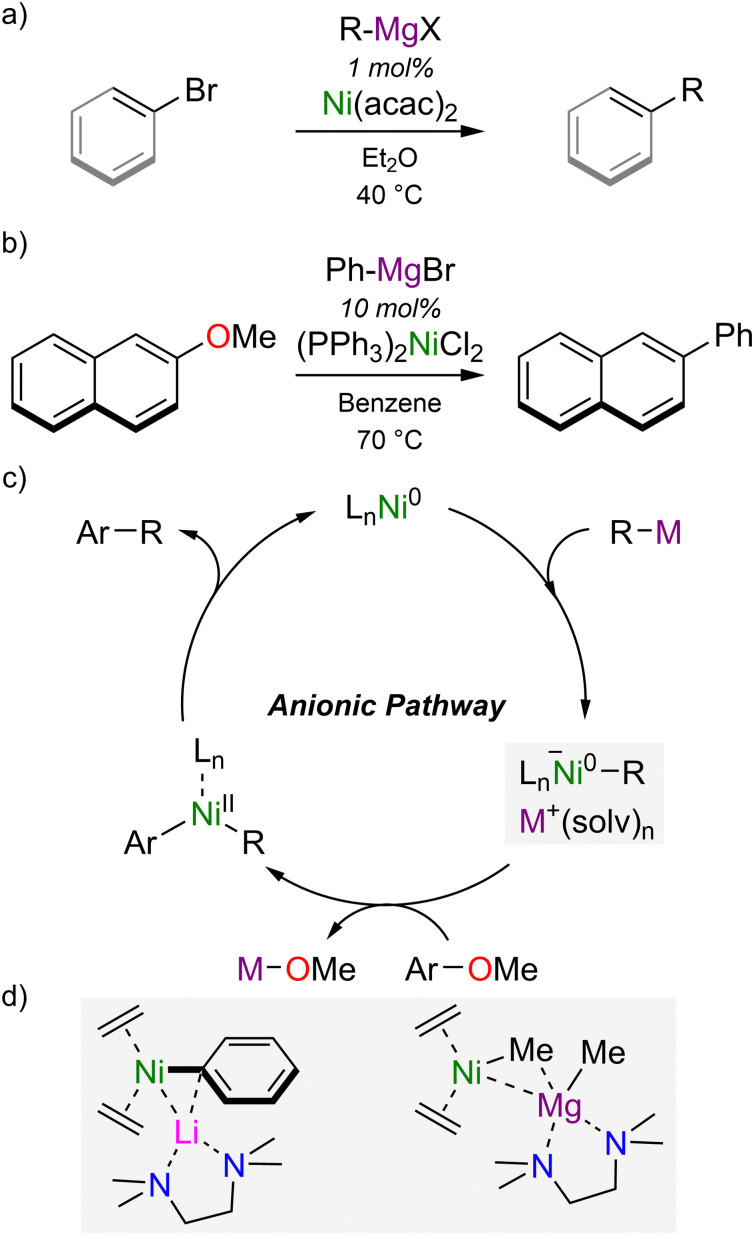
(a) Kumada–Corriu cross-coupling of aryl or vinyl-bromides with Grignard reagents. (b) Ni-catalysed cross-coupling of 2-methoxynaphthalene with PhMgBr (Wenkert reaction). (c) Proposed anionic pathway in the Ni-catalysed cross-coupling of aryl ethers. (d) Isolated and structurally characterised heterobimetallic nickelates proposed as representative intermediates in catalysis.

At a similar time to the discovery of Ni(0)–olefin complexes and heterobimetallic nickelates, the use of nickel complexes in catalytic cross-coupling reactions was independently developed by Kumada and Corriu in 1972 using Ni(acac)_2_ or Ni(ii)–phosphine complexes.^18,19^ Representing one of the earlier transition-metal-catalysed cross-coupling methodologies, the Kumada–Corriu cross-coupling reactions still remains a simple and robust strategy to construct C–C bonds from aryl- or vinyl-halides and Grignard reagents (Scheme 2(a)).^20,21^ Expanding the scope of this transformation, Wenkert reported in 1979 that aryl ethers could serve as electrophilic coupling partners under mild reactions conditions (Scheme 2(b)).^22^ The use of phenol-derived electrophiles (including triflates, esters and aryl ethers) has evolved considerably, particularly in the last two decades, and provides new opportunities for orthogonal cross-coupling strategies or the late-stage functionalisation of decorated aromatics.^23,24^ Mechanistic insights into how nickel facilitates the cross-coupling of aryl ethers lagged behind, however, and the high bond dissociation enthalpy of the C_aryl_–OMe bond raised doubts as to whether classical mechanisms were involved, since oxidative addition would be thermodynamically and kinetically unfavourable, particularly under the mild reactions condition typically employed.^25^ An alternative, “anionic pathway” was proposed by Wang and Uchiyama, who employed DFT calculations to examine the Ni(PCy_3_)_2_-catalysed cross-coupling of anisole with PhM reagents (where M = Li, MgBr or ZnCl), and concluded that heterobimetallic nickelates were the key intermediates that facilitate C_aryl_–OMe bond cleavage.^26,27^

In contrast to classical mechanisms involving Ni(0)/Ni(ii) redox manifolds,^28^ the “anionic pathway” begins by co-complexation of a ligated Ni(0) species with the polar organometallic nucleophile to give a highly reactive nickelate complex that can subsequently undergo oxidative addition and reductive elimination to give the cross-coupled product (Scheme 2(c)).^24–26^ Low-valent heterobimetallic nickelates have subsequently been proposed in a range of other nickel-catalysed transformations involving polar organometallic nucleophiles, including the low-temperature Kumada–Corriu cross-coupling of vinyl bromides,^29,30^ and the silylation of aryl or benzylic ethers.^31–33^ Highly reduced lithium nickelates have also been employed as pre-catalysts for the low-temperature Kumada–Corriu cross-coupling of vinyl bromides, and displayed superior activity to Ni(COD)_2_ or Ni(ii) sources.^29^ Ni(ii)–ate intermediates, on the other hand, have been identified in the multicomponent Ni-catalysed coupling of perfluorinated arenes, Grignard reagents and 1,3-butadiene,^34^ or used directly as catalysts in Kumada–Corriu cross-coupling reactions^35^ or the hydrosilylation of alkenes.^36^ Prior to 2021, the most representative examples of low-valent heterobimetallic nickelates were reported by Cornella and co-workers (Scheme 2(d)),^29,30^ but since these were derived from Ni(0) sources and polar organometallic nucleophiles that differed from those employed under catalytic operating conditions, their precise role in catalysis and the mechanistic insights they provided was limited. Advances in this field therefore necessitated an understanding of contemporary nickel catalysis whilst also revisiting and taking inspiration from early studies into heterobimetallic nickelates.

## 2. Nickelates in catalysis

### 2.1 Cross-coupling of aryl ethers

**Scheme 3:**
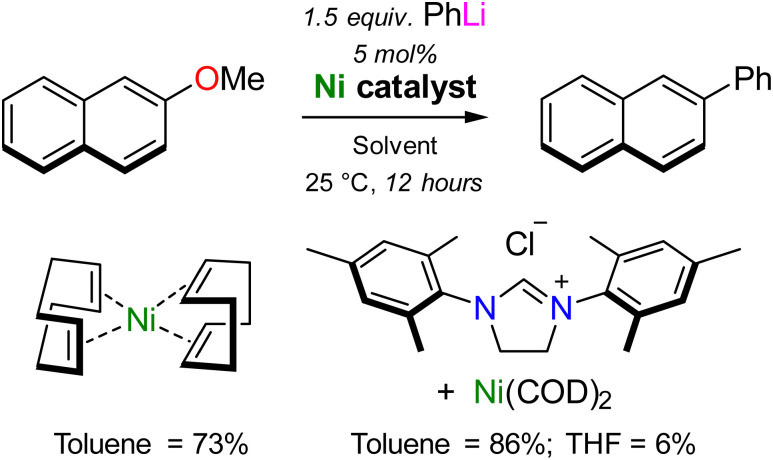
Ligand and solvent dependencies in the Ni-catalysed cross-coupling of 2-methoxynaphthalene with PhLi, as reported by Wang and Uchiyama.^38^

In 2021, Borys and Hevia provided the first experimental support that heterobimetallic nickelates were key intermediates in the Ni-catalysed cross-coupling of aryl ethers.^37^ To ensure that isolated nickelates were directly relevant to catalysis, the Ni(COD)_2_ catalysed cross-coupling of 2-methoxynaphthalene with phenyl-lithium was selected as a model case study. First reported by Wang and Uchiyama in 2016,^38^ two interesting observations were noted (Scheme 3): (i) superior yields (86%) were documented when using N-heterocyclic carbenes as supporting ligands but Ni(COD)_2_ alone still provided the desired product in high yield (73%); (ii) a dramatic solvent influence was apparent with toluene significantly outperforming THF (86% *vs.* 6% yield). The case study therefore aimed to understand how the challenging cross-coupling reaction proceeds in the absence of supporting ligands, and *why* there is a significant solvent influence.

**Fig. 1:**
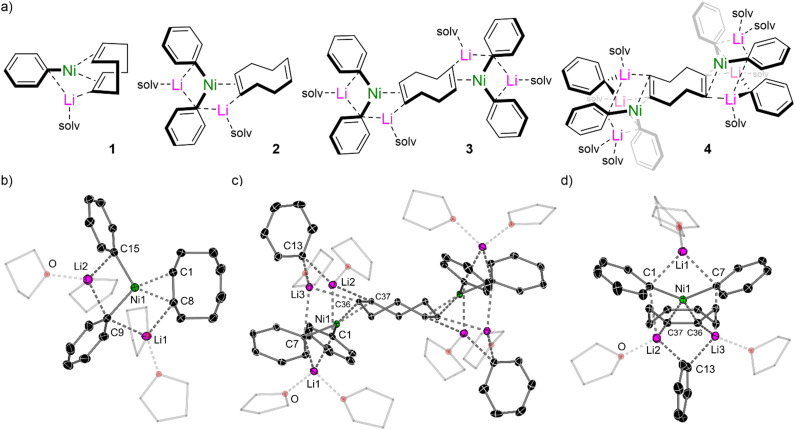
(a) Lithium nickelates (1–4) derived from Ni(COD)_2_ and PhLi. (b) Solid-state structure of Li_2_(THF)_4_Ph_2_Ni(COD) (2). (c) Solid-state structure of [Li_3_(THF)_4_Ph_3_Ni]_2_COD (4). (d) Simplified side-on view of 4 illustrating the co-complexation of additional PhLi within the structure.

No reaction or oxidative addition is observed between Ni(COD)_2_ and 2-methoxynaphthalene, already suggesting that non-classical mechanisms were operative in the absence of supporting ligands. Contrastingly, Ni(COD)_2_ displays rich co-complexation chemistry with PhLi, which is sensitive to the reaction conditions and stoichiometry (Fig. 1(a)). At high concentrations, the 1 : 1 lithium nickelate Li(THF)_2_PhNi(COD) 1, could be observed as a minor species by ^1^H and DOSY NMR spectroscopy, but was found to redistribute to Ni(COD)_2_ and the 2 : 1 lithium nickelate Li_2_(THF)_4_Ph_2_Ni(COD) 2. Different solvates of 2 (including THF, TMEDA and PMDETA) could be readily accessed and fully characterised by multinuclear NMR spectroscopy and single X-ray diffraction. The solid-state structure of 2 (Fig. 1(b)) displays a trigonal-planar Ni-centre bearing two phenyl–carbanionic ligands and one coordinated olefin, in which the C=C (C1–C8) bond is elongated [1.446(2)–1.452(2) Å *vs.* 1.376(5)–1.388(5) Å in Ni(COD)_2_]^39^ due to strong π-back donation. The lithium cations (Li1 and Li2) remain closely contacted to the phenyl-*ipso*-carbons and/or the coordinated olefin ligand. In solution, partial COD dissociation is observed for 2, which affords dinickel complexes with a bridging COD ligand, [Li_2_(THF)_4_Ph_2_Ni]_2_(COD) 3. Different solvates could again be isolated and structurally characterised, and the addition of excess COD was found to push the equilibrium back towards 2. Under catalytic conditions, a large excess of PhLi is present with respect to Ni(COD)_2_ which could lead to the transient formation of higher order species. Treatment of Ni(COD)_2_ with excess PhLi was nevertheless found to give 2 as the major species in THF solution, but a 3 : 1 lithium nickelate, [Li_3_(THF)_4_Ph_3_Ni]_2_COD 4, could also be crystallographically characterised from this reaction mixture. The solid-state structure displays comparable bond metrics and features to 2 and 3, but also contains a third equivalent of PhLi which is co-complexed within the lithium nickelate motif, without direct coordination to Ni (Fig. 1(c) and (d)). This feature has previously been observed in closely-related phenyl–alkali–metal nickelate dinitrogen complexes reported by Krüger, Tsay and Jonas.^14–16^

Whilst the isolation of lithium nickelates derived from Ni(COD)_2_ and PhLi does demonstrate that catalytically relevant heterobimetallic nickelates are synthetically accessible, it does not alone prove that they are directly involved in catalysis. Stoichiometric reactions between *in situ* generated solvates of 2 and 2-methoxynaphthalene (2 equivalents) were performed, which afforded the cross-coupled product (2-phenylnaphthalene) in good yields (60–70%), alongside the corresponding homo-coupling products (biphenyl and 2,2′-binaphthyl) in 10–15% yield each. Reaction monitoring *via* NMR spectroscopy showed that Ni(COD)_2_ is cleanly regenerated after consumption of the lithium nickelate 2 and substrates, illustrating how catalytic turnover could be achieved. Surprisingly, it also revealed that the rate of the cross-coupling reaction is dramatically influenced by the donor solvent present, with Et_2_O and THF solvates of 2 showing immediate conversion (<5 minutes), whilst TMEDA and PMDETA solvates were considerably slower to react (6 days and 12 hours at 25 °C, respectively). This solvent influence is also evident under catalytic conditions, albeit for different reasons (Table 1). Here, the directed *ortho*-lithiation of 2-methoxynaphthalene with PhLi was found to be a competing side reaction that is most favourable when employing strong donors (*e.g.* TMEDA or PMDETA) or when performing the reaction in bulk THF. This is attributed to deaggregation of PhLi into kinetically activated monomers or dimers,^40^ which show enhanced reactivity in deprotonative metalations. This competing side reaction is not observed under stoichiometric conditions, since coordination of PhLi to Ni(COD)_2_ to form the lithium nickelate shuts down its metalating capability. Thus, the optimal conditions for the cross-coupling reaction were found when employing donor-free PhLi in C_6_D_6_, despite its limited solubility (Table 1).

**Table 1:** Ni(COD)_2_ catalysed cross-coupling of 2-methoxynaphthalene with PhLi(solv) to give 2-phenylnaphthalene

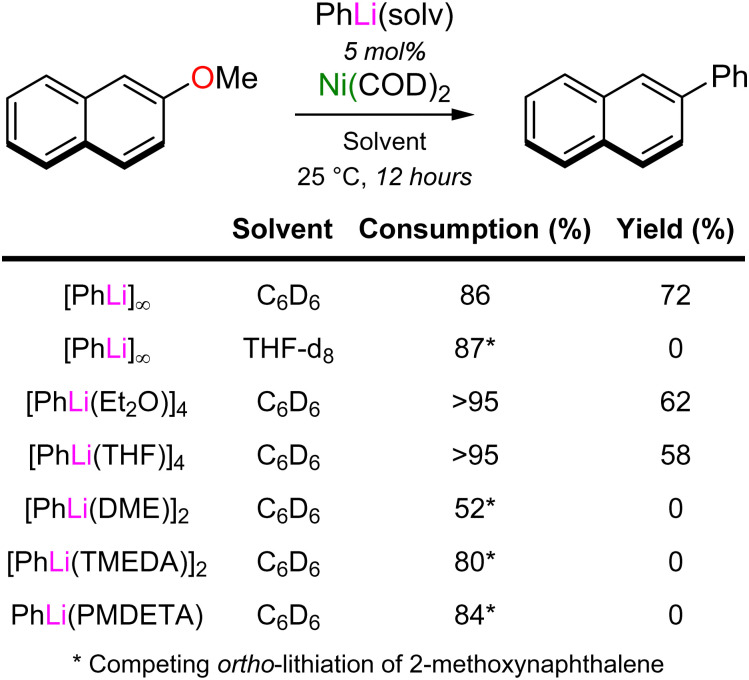

Further support for the involvement of lithium nickelates as key intermediates in catalysis were gained through kinetic studies. This revealed a first-order dependence in Ni(COD)_2_ but zeroth order dependence in both PhLi and 2-methoxynaphthalene, indicating that all three components associate together prior to the rate-limiting oxidation addition and C–OMe bond cleavage. Whilst it was not possible to distinguish between 1 : 1 or 2 : 1 lithium nickelates (1 or 2) due to the zeroth order dependency in PhLi, the stoichiometric studies in tandem with spectroscopic reaction monitoring suggest that 2 : 1 lithium nickelates are the primary on-cycle intermediates. Additionally, isolated lithium nickelates (2, 3 and 4) are all competent catalysts, giving 2-phenylnaphthalene in comparable yields to Ni(COD)_2_. Interestingly, even sub-stoichiometric quantities of donor solvent influenced the rate of the cross-coupling reaction with faster rates observed when using Li_2_(THF)_4_Ph_2_Ni(COD) as a catalyst when compared to Li_2_(TMEDA)_2_Ph_2_Ni(COD). This observation, in tandem with kinetic studies and stoichiometric reactivity, suggested that the ability of the aryl ether to coordinate to the Lewis acidic lithium cation plays a key role in substrate activation, and illustrated that both metals work cooperativity to facilitate this challenging transformation under mild conditions.^37^

**Scheme 4:**
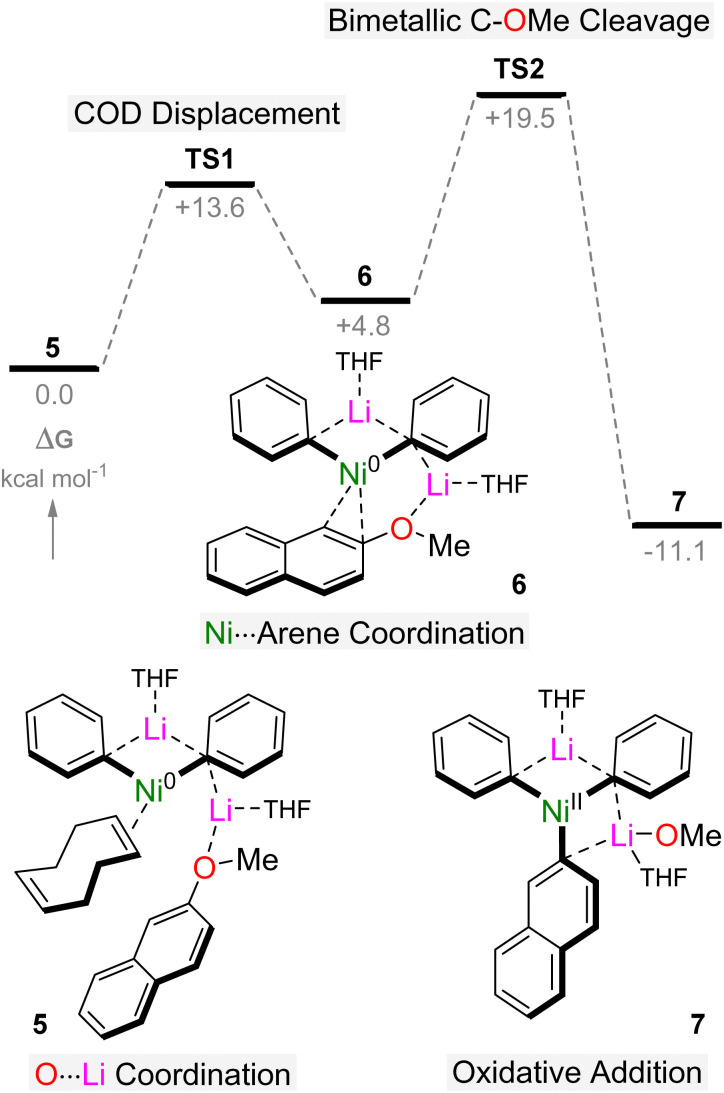
DFT calculated reaction pathway for the oxidative addition of 2-methoxynaphthalene to lithium nickelate 5.

Follow up mechanistic studies into the same model system, in collaboration with Perrin and Payard, were conducted to provide further support for the involvement of lithium nickelates in catalysis and to interrogate later steps of the catalytic cycle.^41^ Starting from the 2 : 1 lithium nickelate 2, which was found to be more energetically favoured compared to the 1 : 1 lithium nickelate 1 when modelled as mono-THF solvates, the reaction begins by coordination of 2-methoxynaphthalene to the Lewis acidic lithium cation to give species 5, which was predicted to be the catalyst resting state based on kinetic studies (Scheme 4). Displacement of COD proceeds with an activation barrier of +13.6 kcal mol^−1^ to deliver intermediate 6 in which the aryl ether is coordinated to the Ni centre in a η^2^-motif. Here, the C–OMe bond is weakened through a combination of back-donation from the electron-rich Ni into the σ* C–O antibonding orbital and coordination of the aryl ether oxygen to lithium. Despite its similarities to Lewis acid assisted mechanisms which have been proposed in the Ni-catalysed cross-coupling of aryl ethers,^25^ no Ni(ii)–OMe bond is formed upon “oxidative addition” of 2-methoxynaphthalene to 6 since LiOMe is concomitantly formed in the reaction. This is noteworthy since Ni(ii)–OMe species have been shown to be unstable intermediates which are prone to β-hydride elimination to give Ni(ii)–H or Ni(0)–CO complexes.^42^ Hence, the “oxidative addition” of 2-methoxynaphthalene to 6 can instead be viewed as a σ-bond metathesis, which delivers Ni(ii) intermediate 7 in which LiOMe is retained within the lithium nickelate structure. The overall barrier for C–OMe bond cleavage from 5 is only +19.5 kcal mol^−1^, consistent with a reaction that proceeds smoothly at room temperature.

**Scheme 5:**
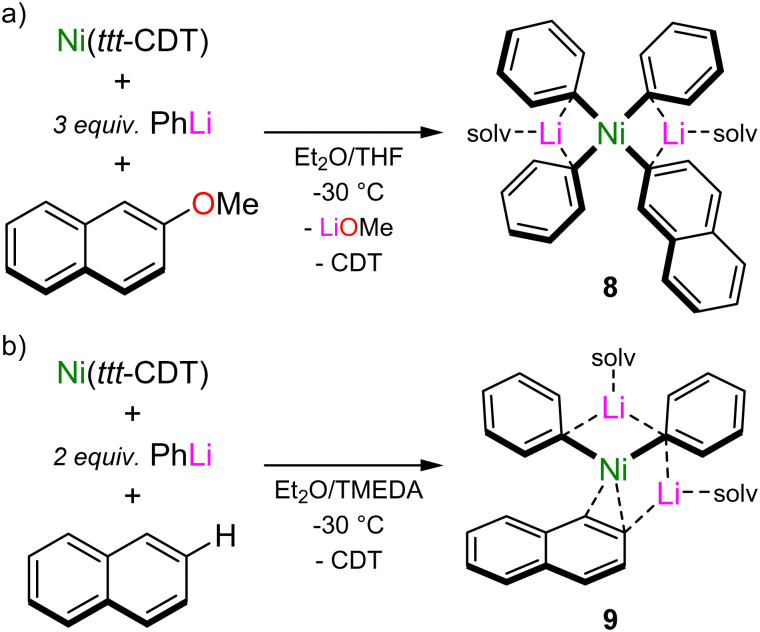
(a) Synthesis of Ni(ii) oxidative addition product, Li_2_(THF)_4_Ph_3_Ni(2-naphthyl) 8. (b) Synthesis of Ni(0) coordination complex, Li_2_(TMEDA)_2_Ph_2_Ni(η^2^-naphthalene) 9.

The calculated reaction pathway reveals that COD dissociation is key to substrate activation and subsequent C–OMe bond cleavage, but since intermediate 6 is endergonic with respect to 5 and 7, its isolation is theoretically impossible. Indeed, under experimental conditions, the treatment of *in situ* generated 2 with 2-methoxynaphthalene leads directly to the cross-coupled product (2-phenylnaphthalene) with clean regeneration of Ni(COD)_2_, and no other intermediates could be isolated or spectroscopically observed.^37^ By switching to Ni(*ttt*-CDT), in which the olefin has been documented to be more labile when compared to COD,^9^ the reaction of Ni(*ttt*-CDT) with 3 equivalents of PhLi and 1 equivalent of 2-methoxynaphthalene at −30 °C affords the square-planar Ni(ii) oxidative addition product, Li_2_(THF)_4_Ph_3_Ni(2-naphthyl) 8 (Scheme 5(a)). This species forms regardless of reaction stoichiometry, indicating that its formation is more favourable over 1 : 1 lithium nickelates. Indeed, displacement of LiOMe from 7 by additional PhLi co-complexation to give 8 was computed to be exergonic by 32.8 kcal mol^−1^. Whilst the proposed intermediate 6 could not be isolated or spectroscopically observed, combining Ni(*ttt*-CDT), PhLi and naphthalene in 1 : 2 : 1 ratio in the presence of TMEDA gave the Ni(0) species, Li_2_(TMEDA)_2_Ph_2_Ni(η^2^-naphthalene) 9 (Scheme 5(b)), which bears striking resemblance to 6. Comparison of the structural and spectroscopic parameters of 6 to analogous phosphine ligated Ni(η^2^-naphthalene) complexes^43^ reveal that the phenyl–carbanion ligands are in fact stronger σ-donors than common neutral ligands, as evidenced by the elongated C=C bond, a feature that has also been theoretically predicted for hypothetical L–Ni(CO)_3_ complexes.^44^ The isolation of compounds 8 and 9 provide strong experimental support that olefin displacement and aryl ether coordination to Ni precedes and indeed facilities oxidation addition and C–OMe bond cleavage.

**Scheme 6:**
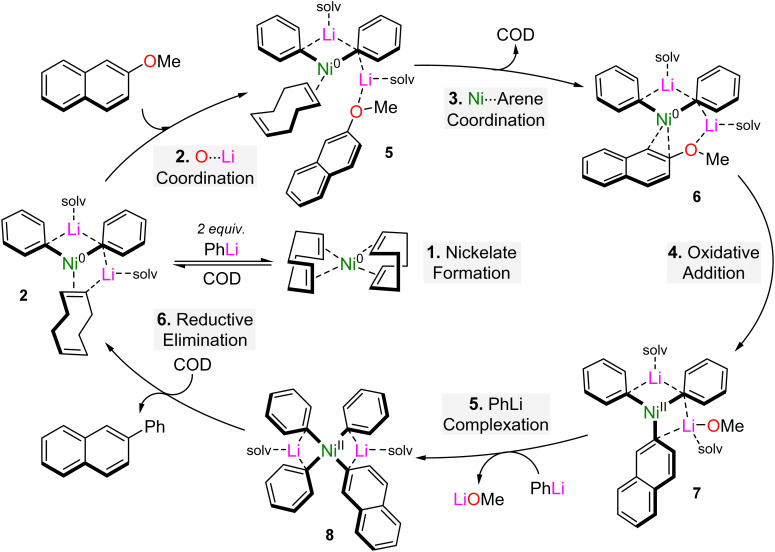
Proposed catalytic cycle for the Ni(COD)_2_ catalysed cross-coupling or 2-methoxynaphthalene with PhLi.

Based on combined experimental and computational insights, a catalytic cycle for the Ni(COD)_2_ catalysed cross-coupling of 2-methoxynaphthalene and PhLi could be constructed (Scheme 6).^41^ This begins by co-complexation of Ni(COD)_2_ with 2 equivalents of PhLi to give 2 : 1 lithium nickelate 2. This can be viewed as an off-cycle equilibrium which reforms Ni(COD)_2_ once PhLi is fully consumed during the reaction. Coordination of 2-methoxynaphthalene to the Lewis acidic lithium cation gives intermediate 5 which undergoes COD dissociation, allowing the substrate to coordinate to Ni in an η^2^-motif 6. This primes the aryl ether for C–OMe bond cleavage which is the rate determining step of the reaction, and affords Ni(ii) intermediate 7. Displacement of LiOMe through further PhLi co-complexation gives the isolable Ni(ii) intermediate 8, which finally undergoes reductive elimination in the presence of COD to deliver the cross-coupled product, 2-phenylnaphthalene, and regenerate the on-cycle 2 : 1 Li/Ni(0) species 2. Experimentally, reductive elimination from 8 occurs smoothly at room temperature in the presence of COD to give 2-phenylnaphthalene in 71% yield, alongside small quantities of biphenyl (9%) and 2,2′-binaphthyl (2%). Computationally, reductive elimination from 8 is also found to be favourable for the cross-coupled product over the homo-coupling products, and proceeds with activation barriers of +16.3 and +17.2 kcal mol^−1^ respectively. Alternative pathways involving the LiOMe by-product were also investigated and found to lower the overall Gibbs energy barrier by 3.2 kcal mol^−1^, but experimental efforts to confirm or rule out the role of LiOMe were inconclusive.

**Scheme 7:**
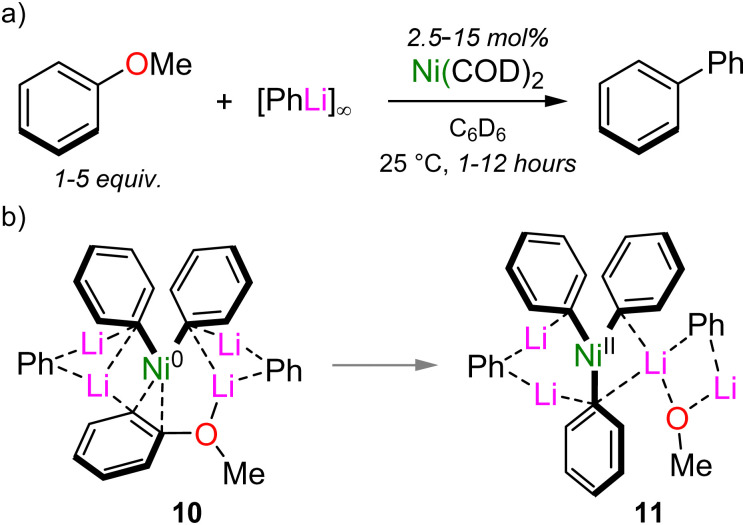
(a) Ni(COD)_2_ catalysed cross-coupling of anisole and PhLi. (b) Proposed lithium nickelate intermediates 10–11.

These complementary studies provide compelling evidence that heterobimetallic nickelates are key intermediates in catalysis, but they also raise the question, “How can this mechanistic knowledge be used help to unlock new reactivity?”. A long-standing problem in nickel catalysis is the so-called “naphthalene problem”,^25^ in which successful electrophiles usually contain multiple fused aromatic rings adjacent to the leaving group. Hence, although non-traditional electrophiles such as aryl ethers can serve as electrophilic coupling partners in Ni-catalysed cross-coupling reactions, it is typically limited to naphthyl derivatives, or those bearing electron-withdrawing substituents.^24^ By taking advantage of solvation, the Ni(COD)_2_-catalysed cross-coupling of anisole with PhLi was also found to be possible by omitting any donor solvents and simply using the aryl ether in sufficient excess (Scheme 7(a)).^41^ This was proposed to have two important consequences: (i) it enables solubilisation of PhLi and any lithium nickelate intermediates; and, (ii) it facilitates the dissociation of COD and η^2^-coordination of anisole to Ni. Experimentally, anisole indeed was found to suitably solubilise PhLi such that transient lithium nickelate intermediates could even be observed by ^1^H NMR spectroscopy. Computationally, the oxidative addition of anisole *via* a similar pathway as proposed for 2-methoxynaphthalene (see Schemes 3 and 5) has an activation barrier of +26.4 kcal mol^−1^, which was deemed too high to account for a reaction proceeding at room temperature. In the absence of coordinating solvents however, higher order lithium nickelate 10 (Scheme 7(b)) was identified as the key intermediate based on its low Gibbs energy and calculated ^1^H NMR spectrum which matched well with those observed experimentally. From here, oxidative addition proceeds with an activation barrier of +16.5 kcal mol^−1^, delivering Ni(ii) intermediate 11.

### 2.2 C–F bond alkynylation

**Scheme 8:**
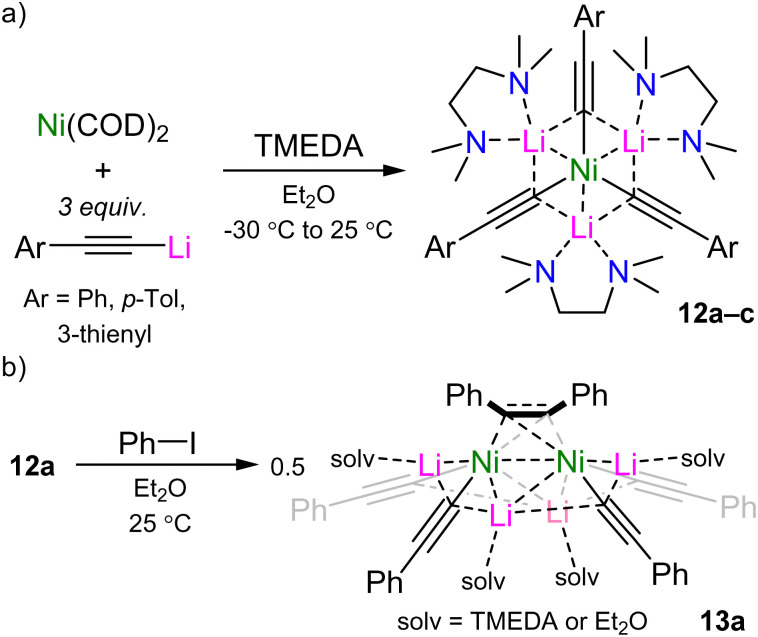
(a) Synthesis of tri-lithium nickelates 12a–c. (b) Stoichiometric reactivity of 12a with iodobenzene to give dinickelate cluster 13a.

The recognition that heterobimetallic nickelates were key intermediates in challenging cross-coupling reactions gave promise that new classes of low-valent nickelates derived from other polar organometallics would also be catalytically competent. In 2022, Hevia and Grabowsky reported a family of homoleptic tri-lithium nickelates derived from Ni(COD)_2_ and aryl-lithium acetylides in the presence of TMEDA (12a–c, Scheme 8(a)).^45^ Whilst this initial work focused on the unique structure and bonding of 12a (see Section 3.2), preliminary studies showed that it reacted stoichiometrically with iodobenzene to give a dinickelate cluster 13a (Scheme 8(b)) in which the diphenylacetylene product, formed through sp–sp^2^ cross-coupling, is coordinated in a μ-η^2^;η^2^-motif between two nickel centres. Compound 13a could also be independently synthesised through the co-complexation of Ni(COD)_2_, Ph–C≡C–Li and Ph–C≡C–Ph in a 2 : 4 : 1 ratio in Et_2_O.

**Fig. 2:**
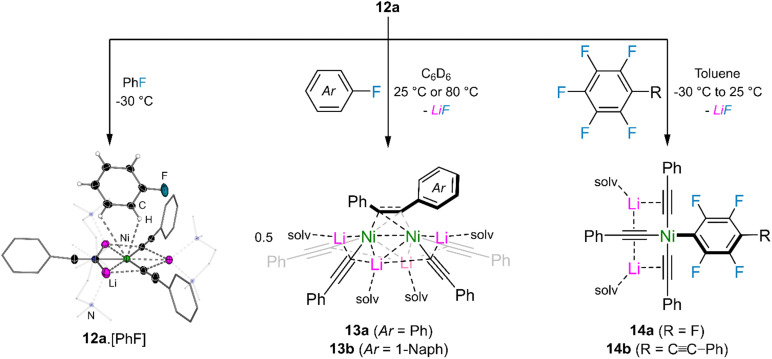
Reactivity of tri-lithium nickelate 12a with aryl fluorides and polyfluorinated arenes.

Whilst no stoichiometric reactivity was observed between 12a and aryl ethers (*e.g.* anisole or 2-methoxynaphthalene), follow up studies in 2024 revealed diverse reactivity with aryl fluorides.^46^ Recrystallisation of 12a from neat fluorobenzene at −30 °C afforded single crystals of the corresponding coordination adduct Li_3_(TMEDA)_3_Ni(C≡C–Ph)_3_·[PhF] 12a·[PhF] in which the fluorobenzene approaches the Ni(0) centre *via* C–H⋯Ni anagostic interactions (Fig. 2, left). This motif is very different to that observed for aryl ethers which adopt η^2^-coordination of the arene to Ni(0) with additional coordination of the ether oxygen to the Lewis acidic lithium cation (see 6 in Schemes 3 and 5).^41^ Trace amounts (<5%) of the cross-coupled dinickelate species 13a are also observed under these neat conditions whilst under stoichiometric conditions, the reaction between 12a and fluorobenzene requires heating to 80 °C for 1 hour. Moving to activated substrates such as 1-fluoronaphthalene enables the reaction to proceed within 15 minutes at room temperature to give the asymmetric species 13b (Fig. 2(b), middle). The reaction of 12a with polyfluorinated arenes follows a different pathway and yields heteroleptic square planar Ni(ii) species 14a–b (Fig. 2, right). This is reminiscent of observations made for aryl ethers (see Schemes 4 and 5)^41^ and suggests that Ni(ii) intermediates such as 14a–b may initially form *en route* to Ni(0) species 13a–b, however this was not directly observed under experimental conditions.

**Scheme 9:**
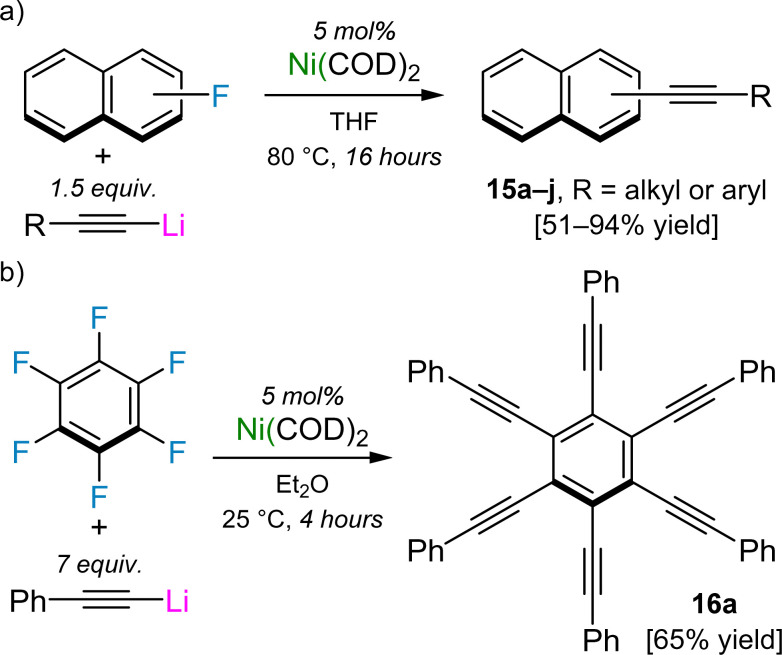
(a) Ni(COD)_2_-catalysed alkynylation of 1- and 2-fluoronaphthalene. (b) Six-fold functionalisation of hexafluorobenzene with Ph–C≡C–Li catalysed by Ni(COD)_2_.

The stoichiometric C–F activation of aryl fluorides and polyfluorinated arenes can also be upgraded to catalytic regimes by simply use Ni(COD)_2_ as a pre-catalyst and lithium acetylides as the nucleophilic coupling partner.^46^ For 1- and 2-fluoronaphthalene, a range of alkyl and aryl substituted lithium acetylides underwent smooth cross-coupling at 80 °C for 16 hours to give the cross-coupled species 15a–j in 51–94% yield (Scheme 9(a)). Less activated substrates such as 4-fluorobiphenyl, fluorobenzene or 4-fluoroanisole do show evidence of cross-coupling, however higher catalyst loadings and elevated reaction temperatures are required, which lead to competing oligomerisation of the formed alkyne products. Remarkably, hexafluorobenzene was found to undergo six-fold functionalisation with Ph–C≡C–Li in just 4 hours at room temperature, to give hexakis(phenylethynyl)benzene 16a in 65% crystalline yield (Scheme 9(b)). 1,4-Difluorobenzene and 1,3,5-trifluorobenzene were also found to undergo di- and tri-functionalisation with Ph–C≡C–Li, albeit with reduced yields (28% and 56% respectively), whilst other polyfluorinated arenes gave intractable mixtures which contained significant quantities of the homo-coupled 1,3-diyne, Ph–C≡C–C≡C–Ph. Although the scope and functional group tolerance of this catalyst system is limited when compared to typical Pd-catalysed Sonogashira cross-coupling reactions,^47,48^ the observation that Ni(COD)_2_ can mediate challenging transformations without the need for additives or external ligands showcases the untapped potential of heterobimetallic nickelates in catalysis.

**Scheme 10:**
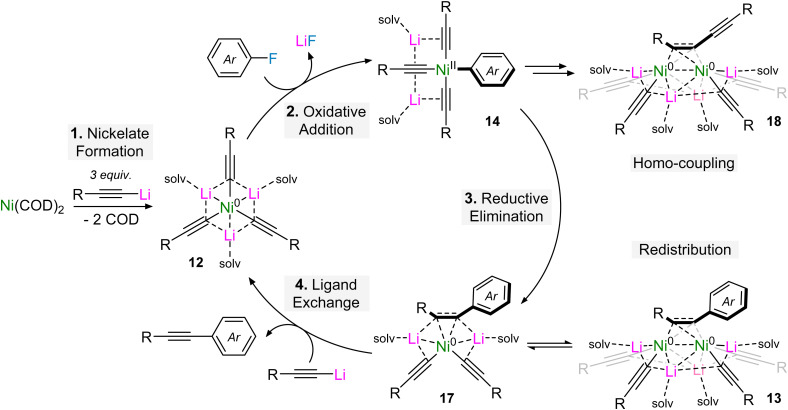
Proposed catalytic cycle for the Ni(COD)_2_-catalysed alkynylation of aryl fluorides and polyfluoroarenes.

Based on stoichiometric and catalytic studies, the Ni(COD)_2_-catalysed alkynylation of aryl fluorides and polyfluorinated arenes was proposed to operate *via* a four-step mechanism.^46^ This begins by co-complexation of Ni(COD)_2_ with three equivalents of the lithium acetylide to give tri-lithium nickelate 12 (Scheme 10).^45^ This then undergoes oxidative addition and concomitant LiF elimination with the aryl fluoride to give square-planar Ni(ii) species 14 which is sufficiently stabilised to be isolated for polyfluorinated arenes but can reductively eliminate to give Ni(0)–alkyne complex 17. Experimentally it was found that the treatment of Li_3_(TMEDA)_3_Ni(C≡C–Ph)_3_12a with fluorobenzene or 1-fluoronaphthalene afforded single crystals of the dinickelate–alkyne species 13a–b, however solution-state reaction monitoring *via* NMR spectroscopy revealed that these reactions first proceed *via*17 which then undergo facile redistribution to give 13. The equilibrium between 13 and 17 could be manipulated by the addition of 12 or the alkyne product but attempts to isolate 17 were unsuccessful – they nevertheless revealed that a competing homo-coupling process was operative, as supported by the isolation of a dinickelate–diyne complex 18. Finally, the catalytic cycle was proposed to close by ligand exchange between 17 and the lithium acetylide, which liberates the cross-coupled alkyne product whilst regenerating the tri-lithium nickelate 12.

### 2.3 Oxidative homo-coupling of lithium acetylides

**Scheme 11:**
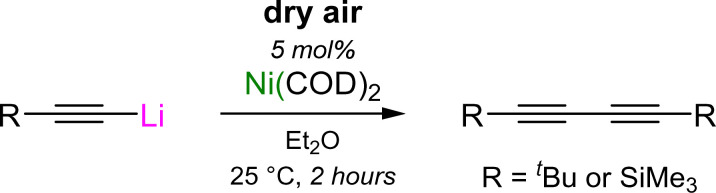
Ni(COD)_2_-catalysed oxidative homo-coupling of lithium acetylides with dry air.

The involvement of lithium nickelates to mediate the formation of C–C bonds can also be extended to the homo-coupling of lithium acetylides to give 1,3-diynes.^49^ Similarly to the cross-coupling of aryl ethers and aryl fluorides, Ni(COD)_2_ can once again be used as a simple pre-catalyst, with dry air now serving as the terminal oxidant (Scheme 11).^50^

**Scheme 12:**
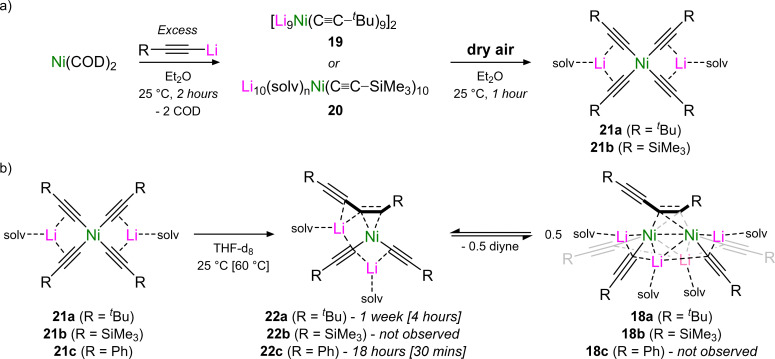
(a) Synthesis of polynuclear lithium nickelate clusters (19–20) and onward aerial oxidation to give homoleptic Ni(ii) complexes 21a–b. (b) Reductive elimination from 21a–c to give nickelate–diyne (22a–c) or dinickelate–diyne complexes (18a–c).

In contrast to the reactivity of Ni(COD)_2_ with aryl lithium–acetylides which yields homoleptic tri-lithium nickelates (see Scheme 7(a) and Section 3.2),^45^ the co-complexation of Ni(COD)_2_ with a large excess of aliphatic lithium–acetylides was found to yield polynuclear lithium nickelate clusters (19–20, Scheme 12(a)) in which 9 or 10 equivalents of organolithium are incorporated per Ni(0) centre (see Section 4.2).^50^ Since the formation of these clusters occurs under catalytically relevant reaction conditions, they were proposed to be the initial species formed upon combining Ni(COD)_2_ with the lithium acetylide in Et_2_O. Upon exposure to dry air, these clusters were observed to oxidise to square-planar homoleptic Ni(ii) species (21a–b) suggesting that a Ni(0)/Ni(ii) redox manifold may be operative, akin to the cross-coupling of aryl ethers and aryl fluorides.^37,41,46^ Supporting this claim, the Ni(ii) species were observed to undergo spontaneous reductive elimination to give Ni(0)–diyne complexes (Scheme 12(b)).^49^ For the ^*t*^Bu derivative 21a, this process was slow at room temperature (1 week) but occurs within four hours at 60 °C to primarily give the mononickelate complex 22a. Similarly to observations made for the cross-coupling of lithium acetylides with aryl fluorides (see Scheme 9),^46^ attempts to isolate 22a were unsuccessful, and consistently yielded the respective dinickelate–diyne complex 18a, even in the presence of excess ^*t*^Bu–C≡C–C≡C–^*t*^Bu. Surprisingly for the SiMe_3_ derivative, no reductive elimination was observed, even with extended heating. Nevertheless, the corresponding dinickelate–diyne complex 18b could be independently prepared through the co-complexation of Ni(COD)_2_, Me_3_Si–C≡C–Li and Me_3_Si–C≡C–C≡C–SiMe_3_ in a 2 : 4 : 1 ratio. Competing ligand exchange processes which initiate through C–Si bond cleavage in the presence of Me_3_Si–C≡C–Li were identified and proposed to be responsible for the low yields observed in the homo-coupling of Me_3_Si–C≡C–Li when compared to the ^*t*^Bu analogue. Contrastingly for the Ph derivative 21c, which could be directly prepared through the reaction of Ni(η^5^-C_5_H_5_)_2_ with four equivalents of Ph–C≡C–Li, reductive elimination was fast (18 hours at 25 °C or 30 minutes at 60 °C) and selectively yielded a mononickelate–diyne complex 22c which could be isolated and fully characterised in the solid-state, and displayed no evidence of redistribution to a dinickelate–diyne species (Scheme 12(b)). The identification of this reductive elimination process, together with the isolation of 22c, helps support mechanistic proposals made for the Ni(COD)_2_-catalysed alkynylation of aryl fluorides (see Scheme 9)^46^ and provides new insights into how heterobimetallic nickelates are involved in specific elementary reaction steps.

### 2.4 Alkyne cyclotrimerisation

**Scheme 13:**
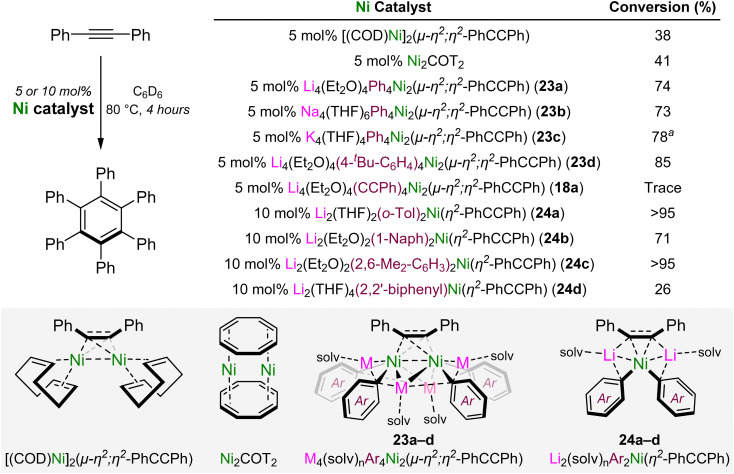
Ni-catalysed [2+2+2] cyclotrimerisation of diphenylacetylene to give hexaphenylbenzene.

A side reaction that was identified in the Ni(COD)_2_-catalysed cross-coupling of aryl fluorides with lithium acetylides was the onward oligomerisation of the cross-coupled alkyne product.^46^ Exemplified by Reppe's pioneering work into the nickel-catalysed oligomerisation of acetylene,^51,52^ numerous well-defined nickel complexes have since been investigated for the catalytic oligomerisation, polymerisation or cycloaddition of substituted alkynes and related unsaturated compounds.^53–55^ In 2023, Borys and Hevia reported a diverse family of diphenylacetylene-coordinated alkali–metal nickelates and assessed their catalytic activity in the [2+2+2] cyclotrimerisation reaction to give hexaphenylbenzene (Scheme 13).^56^

Neutral dinickel complexes, [(COD)Ni]_2_(μ-η^2^;η^2^-Ph–C≡C–Ph)^57^ and Ni_2_COT_2_ (where COT = cyclooctatetraene)^58^ gave poor conversions (38–41%) after heating for 4 hours at 80 °C, whilst alkali–metal dinickelates of the general formula M_4_(solv)_*n*_Ar_4_Ni_2_(μ-η^2^;η^2^-Ph–C≡C–Ph) (23a–d) gave improved conversions (73–85%). These complexes are readily accessed by the co-complexation of Ni(COD)_2_, Ph–C≡C–Ph and the aryl-lithium (PhLi or 4-^*t*^Bu-C_6_H_4_–Li) in a 2 : 1 : 4 ratio, whilst alkali–metal exchange with MO^*t*^Bu (M = Na or K) affords the corresponding sodium or potassium analogues for the Ph derivative. Interestingly, the acetylide derivative Li_4_(Et_2_O)_4_(Ph–C≡C)_4_Ni_2_(μ-η^2^;η^2^-Ph–C≡C–Ph) (18a)^45^ was inactive in the cyclotrimerisation of diphenylacetylene under these reaction conditions but could catalyse the oligomerisation of less challenging terminal alkynes such as phenylacetylene. The use of sterically demanding (*o*-Tol-Li, 1-Naph-Li or 2,6-Me_2_–C_6_H_3_–Li) or geometrically constrained (2,2′dilithiobiphenyl) aryl-lithiums in the co-complexation with Ni(COD)_2_ and Ph–C≡C–Ph afforded mononickelate–alkyne derivatives of the general formaula Li_2_(solv)_*n*_Ar_2_Ni(η^2^-Ph–C≡C–Ph) (24a–d). These complexes were found to be considerably more active in the cyclotrimerisation of diphenylacetylene when compared to the dinickelate–alkyne complexes 23a–d. In addition, it also illustrated how the electronic nature of the carbanionic ligands influences reactivity, with electron-rich substituents outperforming electron-deficient derivatives. The 2,2′-dilithiobiphenyl nickelate complex 24d showed poor catalytic activity in the cyclotrimerisation of diphenylacetylene but was competent for the [4+2] cycloaddition of biphenylene and diphenylacetylene to give 9,10-diphenylphenanthrene.^56^

## 3. Structure and bonding of low-valent nickelates

### 3.1 Dinickelate–benzyne complexes

**Fig. 3:**
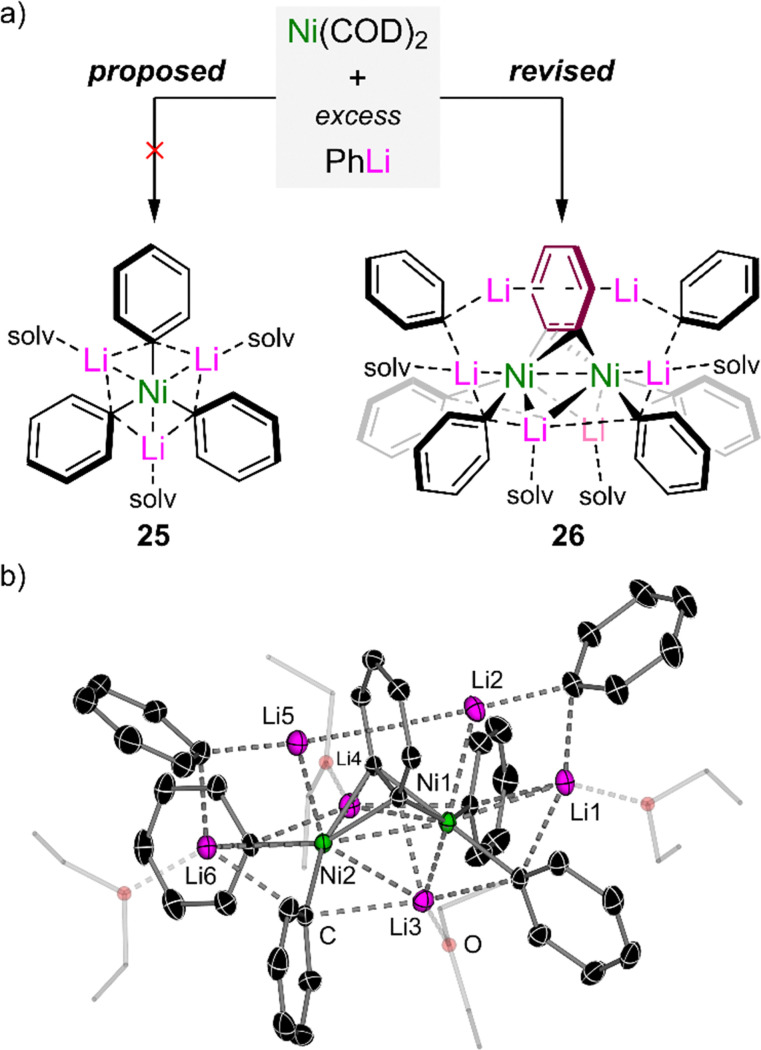
(a) Co-complexation of Ni(COD)_2_ with excess PhLi to give the proposed “Li_3_(solv)_*n*_NiPh_3_” 25 (left) and revised lithium nickelate Li_6_(Et_2_O)_4_Ph_6_Ni_2_(μ-η^2^;η^2^-C_6_H_4_) 26 (right). (b) Solid-state structure of Li_6_(Et_2_O)_4_Ph_6_Ni_2_(μ-η^2^;η^2^-C_6_H_4_) 26.

In 1979, Taube reported that the homoleptic tri-lithium nickelate, “Li_3_(solv)_3_NiPh_3_” 25, could be accessed by the treatment of Ni(COD)_2_ with excess PhLi (Fig. 3(a), left).^59^ The complex was proposed to adopt a planar geometry based on ^13^C NMR spectroscopy, but the lack of a solid-state structure raised questions about the potential bonding situation in this unique complex. Seeking to find answers to this forty-year-old mystery, the Hevia and Campos groups independently assessed the co-complexation of Ni(COD)_2_ with PhLi under various conditions and ultimately concluded that 25 had been structurally misassigned.^60^ Crystallographic and spectroscopic studies unambiguously revealed that a benzyne-type complex of the formula Li_6_(Et_2_O)_4_Ph_6_Ni_2_(μ-η^2^;η^2^-C_6_H_4_) 26 was instead formed under these conditions (Fig. 3(a), right), suggesting that “Li_3_(solv)_3_NiPh_3_” is too unstable to be formed at all or to be isolated under ambient conditions.

The solid-state structure of 26 (Fig. 3(b)) reveals a dinickelate motif in which a central C_6_H_4_ ligand bridges between two nickel centres. Two molecules of PhLi are formally coordinated to each Ni, whilst two additional molecules co-complex within the structure without direct interaction with nickel. Both of these features are reminiscent of alkali–metal nickelate–dinitrogen complexes reported by Krüger, Tsay and Jonas,^14–16^ prepared through the addition of excess PhLi (or PhNa) to Ni(*ttt*-CDT) under an N_2_ atmosphere. The formation of 26 indicates that “Li_3_(solv)_3_NiPh_3_” is too electron-rich to be stable,^59^ leading to the *in situ* generation of a π-accepting benzyne ligand to modulate the electron density at nickel. The benzyne-type ligand was proposed to form through “LiH” elimination from PhLi but attempts to verify this experimentally were inconclusive. Despite the similar structural features to μ-η^2^;η^2^-N_2_ (N≡N)^14–16^ and μ-η^2^;η^2^-alkyne (R–C≡C–R) complexes,^45,46,56^ bonding analysis *via* DFT calculations on 26 indicate that the benzyne ligand is best described as a [C_6_H_4_]^2−^ core with formally Ni(i) centres – this is reinforced by the very long C–C bond length of 1.449(6) Å which is considerably longer than genuine Ni–benzyne complexes.^61^ Natural bond order (NBO) analysis on 26 reveals that backdonation from filled Ni d-orbitals to the π*-orbital of the C_6_H_4_ ligand is the strongest bonding interaction, with a stabilisation energy of 474.1 kcal mol^−1^. The coordination of the phenyl–carbanion lone pairs to the empty s-orbital of Ni provides σ-donation amounting to ∼50 kcal mol^−1^ each, whilst π-donation from the C_6_H_4_ π-system also makes a significant contribution (29.3 kcal mol^−1^). This highlights that push–pull stabilisation (*i.e.* a fine balance of donor and acceptor bonding interactions) is essential for the construction and isolation of low-valent heterobimetallic nickelates.

### 3.2 Homoleptic tri-lithium nickelates

**Fig. 4:**
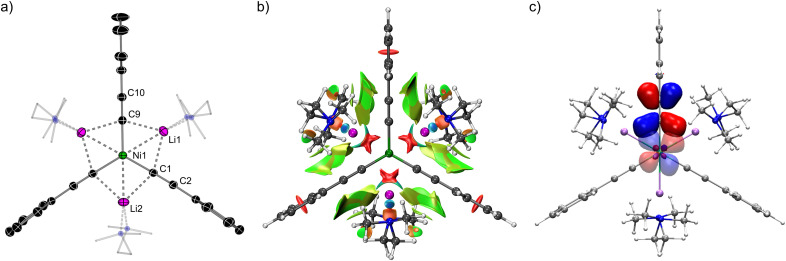
(a) Solid-state structure of Li_3_(TMEDA)_3_Ni(C≡C–Ph)_3_12a. (b) Isosurface representation of the non-covalent interactions (NCI index) where red = repulsive; blue = attractive; green = weakly attractive. (c) Natural bond orbitals showing overlap of the Ni d-orbital with the C≡C π*-orbital.

Prior to 2022, all documented low-valent heterobimetallic nickelates were derived from alkyl (sp^3^) or aryl (sp^2^) carbanions, and studies into the co-complexation of Ni(0)–olefins with metal acetylides (sp) were unknown. The NBO analysis conducted on 26 (see Section 3.1)^60^ predicted that acetylides would serve as ideal partners for Ni(0) due to greater s-orbital overlap, whilst also acting as built-in π-acceptors to modulate the high electron density. Indeed, the combination of Ni(COD)_2_ with three equivalents of aryl lithium–acetylides in the presence of TMEDA (see Scheme 7) affords Li_3_(TMEDA)_3_Ni(C≡C–Ar)_3_12a–c, the first examples of homoleptic tri-lithium nickelates.^45^ The solid-state structure of 12a (Ar=Ph, Fig. 4(a)) displays a perfectly planar environment around Ni with Ni⋯Li distances ranging from 2.487(4)–2.512(3) Å – this is within the sum of covalent radii (2.52 Å)^62^ and comparable to other structurally characterised lithium nickelates.^41,45,46,50,56,60^

**Scheme 14:**
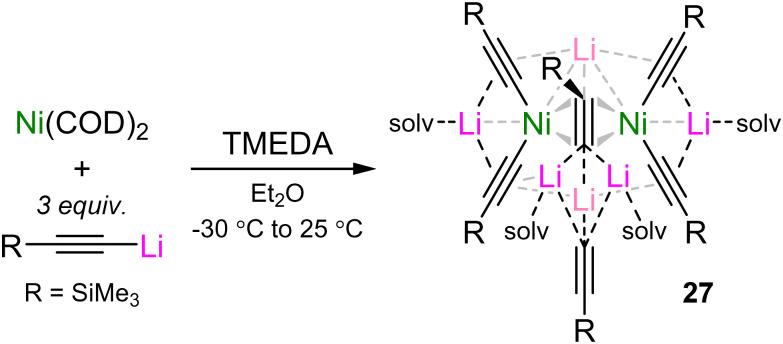
Synthesis of dinickelate complex 27 through the co-complexation of Ni(COD)_2_ and Me_3_Si–C≡C–Li in the presence of TMEDA.

This observation implied that 12a may contain the very rare hexagonal planar geometry,^63,64^ however subsequent complementary bonding analysis disproved this initial hypothesis.^45^ Specifically, a plot of the non-covalent interactions (NCI, Fig. 4(b)) revealed that any interaction between Ni and Li is repulsive in nature, as indicated by the red isosurface. In addition, the quantum theory of atoms in molecules (QTAIM) analysis showed no bond critical point (bcp) between Ni and Li, supporting that there is no covalent metal–metal bonding. The NCI plot nevertheless displayed weakly attractive London dispersion interactions between the TMEDA ligand and acetylide π-system, as indicated by the green isosurface. Experimentally, TMEDA was found to crucial for the stabilisation and thus isolation of 12a–c, since attempts to access these complexes in the absence of TMEDA or presence of other donor solvents were unsuccessful. Supporting the claim that acetylides would serve as ideal partners for Ni(0), the NBO analysis on 12a indicated that σ-donation from the carbanion lone pair amounts to 68 kcal mol^−1^ (*cf.* ∼50 kcal mol^−1^ for Ph → Ni), whilst back-donation from Ni(0) in plane d-orbitals (d_*xy*_ and d_*x*^2^−*y*^2^_) to the C≡C π* amounts to 11.5 kcal mol^−1^ each (Fig. 4(c)). In addition, there is also back-donation from the out-of-plane d_*xz*_ and d_*yz*_ orbitals to the orthogonal C≡C π*-orbital, alongside a weak interaction from the Ni d_*z*^2^_ to C≡C σ*-orbital. Extending this synthetic strategy to Me_3_Si–C≡C–Li under identical reaction conditions did not afford the corresponding tri-lithium nickelate, but instead afforded a dinickelate species in which one molecule of the acetylide is coordinated in a μ-η^2^;η^2^-motif (akin to related benzyne, alkyne and N_2_ complexes)^14–16,45,46,56,60^ whilst one molecule of lithium acetylide co-complexes within the structure without direct interaction with Ni(0) (Scheme 14). This suggested that the aliphatic lithium acetylide is too electron-rich to form a stable tri-lithium nickelate, and demonstrates the structural flexibility that alkali–metal nickelates can adopt in order to accommodate surplus electron density.

## 4. Coordination and Co-complexation chemistry of alkali–metal nickelates

### 4.1 Coordination of unsaturated organic π-acceptors

**Scheme 15:**
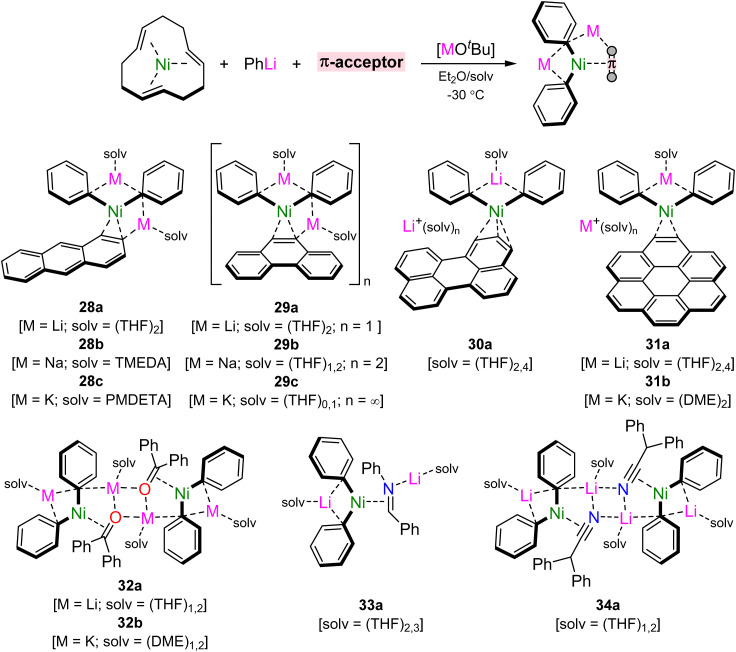
Synthesis of 2 : 1 phenyl–alkali–metal nickelates bearing different π-accepting ligands.

Mechanistic studies into the role of lithium nickelates in challenging cross-coupling reactions revealed that the ability for the substrate to π-coordinate to Ni(0) preceded oxidative addition,^41^ whilst the quest to obtain a homoleptic tri-lithium nickelate illustrated that push–pull stabilisation is essential to prepare and isolate low-valent heterobimetallic nickelates.^45,60^ Building on from these themes and seeking to establish fundamental knowledge into these under explored complexes, Borys and Hevia recently explored the rich coordination chemistry of 2 : 1 phenyl–alkali–metal nickelates with a diverse series of organic π-accepting ligands.^65^ These can be readily prepared through the combination of Ni(*ttt*-CDT) with two equivalents of PhLi followed by ligand exchange with suitable π-accepting ligands and alkali–metal exchange with MO^*t*^Bu (M = Na or K) in the presence of suitable donor solvents or ligands (Scheme 15). This methodology firstly granted access to homologous series of lithium, sodium and potassium nickelates containing η^2^-anthracene (28a–c) and η^2^-phenanthrene (29a–c) ligands. In the former case, monomeric complexes were obtained by differing the donor ligand to match size of the alkali–metal cation, whilst in the latter case, keeping the THF solvent consistent gave monomeric (Li), dimeric (Na) and polymeric (K) motifs in the solid-state, reflecting the diverse coordination preferences of these heterobimetallic systems. Extending the conjugation of the π-accepting ligand to perylene and coronene gave isostructural *pseudo*-solvent separated ion pairs, [Li(THF)_2_Ph_2_Ni(π-ligand)][Li(THF)_4_], 30a and 31a. Attempts to prepare sodium and potassium analogues of 30a led to single electron reduction and the isolation of perylene radical anions,^66^ illustrating the highly reducing nature of the alkali–metal nickelates (*cf.* one-electron reduction potential of perylene = −1.98 V).^67^ Contrastingly, heavier alkali–metal nickelates could be accessed for the coronene complex (*cf.* one-electron reduction potential of coronene = −2.36 V),^67^ which gave K_2_(DME)_4_Ph_2_Ni(η^2^-coronene) (31b) as a contacted species.

Replacing the all-carbon polyaromatic hydrocarbons for heteroatom containing π-accepting ligands further expanded the scope of alkali–metal nickelates.^65^ Ligand exchange with benzophenone, *N*-benzylideneaniline and diphenylacetonitrile was successful, despite the possibility of competing reduction, nucleophilic addition and deprotonation reactions by Ni(0) or the organometallic reagent. For benzophenone 32a and diphenylacetonitrile 34a, isostructural dimeric motifs were obtained, whilst *N*-benzylideneaniline yielded a monomeric species 33a. The former could also be extended to the potassium analogue 32b, which similarly gave a dimeric motif solvated by DME. In all cases, the solid-state structures displayed significantly elongated C=C, C=O, C=N or C≡N bonds, whilst the ^1^H and/or ^13^C NMR spectra showed drastically upfield shifted (shielded) resonances for coordinated sites. These features demonstrate the very strong back-donation which is present in the systems, which far exceeds that observed in related phosphine or N-heterocyclic carbene Ni(0) complexes. The diverse coordination chemistry presented in these heterobimetallic systems, alongside their emerging role in catalysis, presents new opportunities for challenging bond activation and functionalisation.

### 4.2 Polynuclear clusters

A common observation in the preparation of lithium nickelates is the ability for additional molecule of organolithium to co-complex within the heterobimetallic motif without direct coordination to Ni(0) (*e.g.* see complexes 4, 26 and 27).^37,45,60^ This feature typically emerges through the treatment of Ni(0)–olefins with a large excess of the organometallic nucleophile, which imitates catalytic conditions, but ultimately originates from the aggregation and solvation of organolithium itself.

**Fig. 5:**
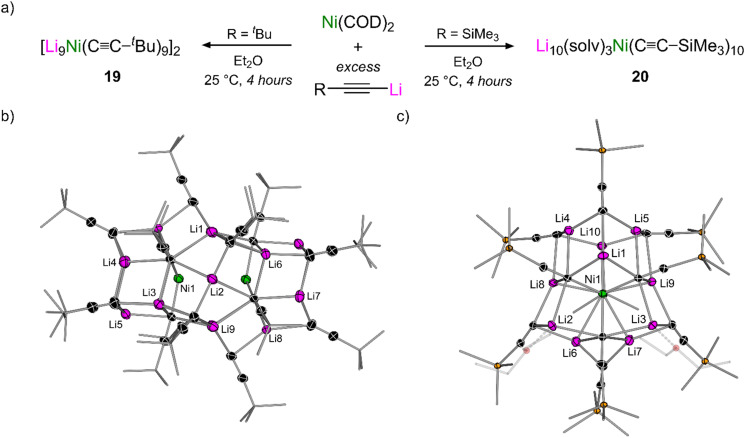
(a) Synthesis of polynuclear lithium nickelate clusters 19–20. (b) Solid-state structure of [Li_9_Ni(C≡C–^*t*^Bu)_9_]_2_ (19). (c) Solid-state structure of Li_10_(Et_2_O)_3_Ni(C≡C–SiMe_3_)_10_ (20).

Examples of 1 : 1, 2 : 1 and 3 : 1 lithium nickelates have been documented in early and more recent studies,^9,17^ and this can also be extended to 9 : 1 and 10 : 1 systems, giving polynuclear lithium nickelate clusters.^50^ The treatment of Ni(COD)_2_ with excess ^*t*^Bu–C≡C–Li afforded [Li_9_Ni(C≡C–^*t*^Bu)_9_]_2_ (19, Fig. 5(a), left) whilst the use of Me_3_Si–C≡C–Li gave Li_10_(Et_2_O)_3_Ni(C≡C–SiMe_3_)_10_ (20, Fig. 5(a), right). The solid-state structure of 19 (Fig. 5(b)) displays a solvent-free 20-metal cluster containing two tri-lithium nickelate Li_3_Ni(C≡C–^*t*^Bu)_3_ distorted planes, two cyclo-trimeric lithium acetylide “end-caps” and a bridging cyclo-hexameric lithium acetylide core. Contrastingly, the solid-state structure of 20 (Fig. 5(c)) is an 11-metal cluster which contains a tetrahedral tetra-lithium nickelate Li_4_Ni(C≡C–SiMe_3_)_4_ core flanked by six additional molecules or organolithium, three of which are solvated by Et_2_O. The isostructural ^*t*^Bu analogue of 20 could also be identified by changing the crystallisation solvent from pentane to a mixture of Et_2_O and (Me_3_Si)_2_O. ^1^H Diffusion order spectroscopy (DOSY) NMR studies revealed that both clusters are fully retained in non-donor solvents (toluene-d_8_) whilst in donor solvents (THF-d_8_) they deaggregate to their tri-lithium nickelate (for 19) or tetra-lithium nickelate cores (for 20) respectively. DOSY studies in tandem with X-ray crystallography helped to provide an explanation for the different clusters that are formed for seemingly similar organolithiums – ^*t*^Bu–C≡C–Li forms decameric aggregates in weakly coordinating solvents whilst Me_3_Si–C≡C–Li forms hexameric aggregates.^49,50^ The hypothesis that organolithium aggregation serves as a “blueprint” in the construction of the polynuclear lithium nickelate clusters was supported by the finding that the clusters do not form (and are not retained) in strong donor solvents such as THF.

## 5. Conclusions & outlook

This Feature Article showcases recent developments in the field of alkali–metal nickelates, highlighting their emerging applications which span mechanistic investigations, sustainable catalysis and fundamental structure, bonding, and reactivity studies. The active role of low-valent nickelates in the cross-coupling of aryl ethers and aryl fluorides has provided new insights into how nickel can mediate challenging transformations in the absence of supporting ligands and the key roles played by the alkali–metal and solvents. The quest to obtain a homoleptic tri-lithium nickelate has revealed diverse structural motifs and provided essential fundamental insights into the unique bonding and push–pull stabilisation of low-valent heterobimetallic nickelates. The rich coordination ability and co-complexation chemistry of alkali–metal nickelates has also been disclosed and these features hold promise for substrate binding and catalyst activation strategies towards the functionalisation of small molecules and strong bonds. The mechanistic knowledge gained through these studies, and recognition that such heterobimetallic complexes exhibit potent catalytic reactivity in the absence of traditionally employed external ligands, provides a blueprint for the development of sustainable catalysis. We anticipate that future studies will continue to discover and leverage the involvement of low-valent nickelates in other catalytic transformations and believe that other metalates beyond nickel should also be considered as viable intermediates in reactions employing polar organometallics.

## Data availability

No new data was generated or analysed in this Feature Article.

## Conflicts of interest

There are no conflicts to declare.
